# The moral psychology of rationing among physicians: the role of harm and fairness intuitions in physician objections to cost-effectiveness and cost-containment

**DOI:** 10.1186/1747-5341-8-13

**Published:** 2013-09-08

**Authors:** Ryan M Antiel, Farr A Curlin, Katherine M James, Jon C Tilburt

**Affiliations:** 1 Department of Surgery, Mayo Clinic, Rochester, Minnesota, USA; 2 Program in Professionalism and Ethics, Mayo Clinic, Rochester, Minnesota, USA; 3 Department of Medicine and the MacLean Center for Clinical Medical Ethics, University of Chicago, Chicago, Illinois, USA; 4 Biomedical Ethics Research Unit, Mayo Clinic, Rochester, Minnesota, USA; 5 Division of General Internal Medicine, Mayo Clinic, Rochester, Minnesota, USA; 6 Knowledge and Evaluation Research Unit, Mayo Clinic, Rochester, Minnesota, USA

**Keywords:** Physicians, Survey, Moral beliefs, Cost-effectiveness, Cost-containment

## Abstract

**Introduction:**

Physicians vary in their moral judgments about health care costs. Social intuitionism posits that moral judgments arise from gut instincts, called “moral foundations.” The objective of this study was to determine if “harm” and “fairness” intuitions can explain physicians’ judgments about cost-containment in U.S. health care and using cost-effectiveness data in practice, as well as the relative importance of those intuitions compared to “purity”, “authority” and “ingroup” in cost-related judgments.

**Methods:**

We mailed an 8-page survey to a random sample of 2000 practicing U.S. physicians. The survey included the MFQ30 and items assessing agreement/disagreement with cost-containment and degree of objection to using cost-effectiveness data to guide care. We used t-tests for pairwise subscale mean comparisons and logistic regression to assess associations with agreement with cost-containment and objection to using cost-effectiveness analysis to guide care.

**Results:**

1032 of 1895 physicians (54%) responded. Most (67%) supported cost-containment, while 54% expressed a strong or moderate objection to the use of cost-effectiveness data in clinical decisions. Physicians who strongly objected to the use of cost-effectiveness data had similar scores in all five of the foundations (all p-values > 0.05). Agreement with cost-containment was associated with higher mean “harm” (3.6) and “fairness” (3.5) intuitions compared to “in-group” (2.8), “authority” (3.0), and “purity” (2.4) (p < 0.05). In multivariate models adjusted for age, sex, region, and specialty, both “harm” and “fairness” were significantly associated with judgments about cost-containment (OR = 1.2 [1.0-1.5]; OR = 1.7 [1.4-2.1], respectively) but were not associated with degree of objection to cost-effectiveness (OR = 1.2 [1.0-1.4]; OR = 0.9 [0.7-1.0]).

**Conclusions:**

Moral intuitions shed light on variation in physician judgments about cost issues in health care.

## Introduction

How best to cut health care spending has been central to debates about health care reform [1]. Analysts predict that if current trends continue, the United States will spend approximately 38% of its gross domestic product on health care by 2075 [2], a state that health economists believe is unsustainable [3]. Comparative effectiveness research (CER), as defined by the Agency for Healthcare Research and Quality, “is designed to inform health-care decisions by providing evidence on the effectiveness, benefits, and harms of different treatment options [4]. Proponents hope that CER could help slow the rise of health care costs through more prudent application of evidence to care. Amid discussions on how to implement CER, prominent professional societies such as the American College of Physicians have called for comparative effectiveness research to include cost-effectiveness analysis [5]. In 1996, the U.S. Panel on Cost-Effectiveness in Health and Medicine proposed that cost-effectiveness analyses should use quality-adjusted life-years (QALYs) to assign value to health care outcomes [6]. However, critics have argued that current proposals for using cost-effectiveness analysis go too far and become veiled attempts to ration health care by cutting provider reimbursements [7].

Although these disputes have been carried out largely in the arena of health policy, practicing physicians will have a lot to say about how CER and cost-effectiveness analysis are used to guide health care decisions in the clinical arena [8-10]. Some argue that physicians have a civic duty to balance the needs of society and the needs of the individual patient [11,12]. Yet, “bedside rationing” has been criticized for being exclusively utilitarian [13] and at odds with multi-faceted conceptions of justice [14-17]. Moreover, many physicians feel that their foremost professional obligation is to advocate for their individual patients, without regard to the broader questions regarding cost and resources [18]. To date, there has been little empirical research that might begin to explain why some physicians embrace CER and cost-effectiveness analysis and others resist them.

Social and cognitive psychology have recently generated novel approaches for defining basic differences in moral intuitions. Social-intuitionist theory, in particular, posits that ideological divides do not arise from debates over *moral reasoning* but rather over differences in the innate or ‘gut’ instincts about morality called *moral foundations*. The theory proposes that these moral foundations have arisen in different cultures to build social collaboration [19]. Haidt has identified five different moral foundations: harm/care, fairness/reciprocity, ingroup/loyalty, authority/respect, and purity/sanctity [19]. Moral disagreements are often explained by differences in the relative weight that opposing parties place on each of the five foundations. For instance, political liberals construct their moral judgments preferentially on intuitions of *harm* and *fairness*, while political conservatives judge morality with more or less equal shares of the five foundations [20]. Haidt has found that these constructs explain differences in moral judgments on a range of issues [21].

This study examines whether a social intuitionist theoretical framework may explain differences in physicians’ judgments about using cost-effectiveness data to guide clinical decisions as well as their judgments about other cost-containment strategies. We hypothesized that harm and fairness ratings would be directly associated with favorable perceptions of using cost-effectiveness data and cost-containment strategies [20,21].

## Methods

### Sample and procedures

In May 2009, we mailed a confidential, self-administered questionnaire to 2000 practicing U.S. physicians ages 65 and under from all specialties. Our random sample of physicians was selected from the AMA Masterfile, a database devised to include virtually all U.S. physicians. The initial mailing included a book as a gift and promised an additional $25 to all respondents. Physicians who did not respond to the first mailing were sent up to two subsequent mailings. The Mayo Clinic Institutional Review Board approved this study.

### Primary measures

The details of the survey’s development and implementation have been published elsewhere [22]. We asked physicians to what extent they agreed with limiting reimbursements for expensive drugs and procedures in order to expand coverage to uninsured patients (cost-containment). [specific wording of items is included in results tables] Answers to this item were on a 4-point ordinal response scale. We also asked respondents to rate their degree of moral objection (none, moderate, or strong) to using cost-effectiveness data in clinical decisions. These measures were cognitively tested with eight practicing physicians for clarity, balance, and ease of response categories during a pilot phase of the survey.

The primary predictors were physicians’ scores on the Moral Foundations Questionnaire (MFQ30) – a measure of the five key foundations of social intuitionism (*harm/care, fairness/reciprocity, ingroup/loyalty, authority/respect, and purity/sanctity*) [23]. Physicians’ mean scores for the five moral foundations were calculated based on their responses to six survey items for each foundation. The MFQ30 contains two parts, the first of which measures the degree of agreement or disagreement with various statements. Each of these items were scored on a scale ranging from 1 to 6, with 1 being “strongly disagree”, 2 being “moderately disagree”, 3 being “slightly disagree”, 4 being “slightly agree”, 5 being “moderately agree”, and 6 being “strongly agree”. The second part of the MFQ30 examines the relevance of various items in determining whether or not something is right or wrong. These items were scored on a scale ranging from 1 to 6, with 1 being “not at all relevant”, 3 being “somewhat relevant”, and 6 being “extremely relevant” to determining whether or not something is right or wrong.

### Analysis

All data were double entered and 100% verified. We obtained descriptive statistics (including mean, standard deviation, and range) for the five moral foundations and calculated raw Cronbach’s alpha scores for each subscale to assess the internal consistency of individual survey items comprising each subscale. For simplicity of presentation, we then dichotomized our primary criterion variables of interest (any objection vs. no objection to using cost-effectiveness in clinical decisions; and agreement vs. disagreement with cost-containment) and used simple and multiple logistic regression to examine associations between harm and fairness subscale scores and judgments about cost-effectiveness and cost-containment. Multiple regression models included age, sex, region, and specialty. All analyses were conducted using SAS, version 9.1 (Cary, NC). The funding source had no role in study design, implementation or analysis.

## Results

1032 of 1895 physicians (54%) responded (105 could not be contacted). Characteristics of respondents are shown in Table 1.

**Table 1:** Characteristics of survey respondents for whom demographic data were available

**Characteristic**	**No./Total No. (%)**
Female sex	283/1011 (28)
Age (years)	
Less than 50	471/1011 (47)
50 or older	540/1011 (53)
Race or ethnic group	
White or Caucasian	786/1011 (78)
Asian	146/1011 (14)
Other	50/1011 (5)
Black or African-American	25/1011 (2)
American Indian or Alaska	4/1011 (0.4)
Native	
Region*	
South	331/1032 (32)
Midwest	251/1032 (24)
Northeast	227/1032 (22)
West^†^	215/1032 (21)
Primary specialty	
Primary care	407/1032 (39)
Surgery	212/1032 (21)
Procedural specialty	206/1032 (20)
Nonprocedural specialty	175/1032 (17)
Non-clinical	22/991 (2)
Other	10/991 (1)

*8 responding physicians were from Puerto Rico.

^†^ Includes 6 physicians from Hawaii and 3 from Alaska.

Physicians were split in their views on using cost-effectiveness data to determine which treatments will be offered to patients: 14% expressed a strong moral objection, 40% reported a moderate objection, and 45% expressed no moral objection. As reported elsewhere [22], most respondents (67%) supported limiting reimbursement for expensive drugs and procedures if that would result in expanded access to basic health care for those currently without such care (Table 2).

**Table 2:** Distribution of physicians’ responses to items on cost-containment strategies and cost-effectiveness data, as well as physicians’ overall mean scores for the five constructs of moral foundations

**Survey item and response options**	**N (%)**
**Cost-containment**	
I would favor limiting reimbursement for expensive drugs and procedures if that would help expand access to basic healthcare for those currently lacking such care	
Strongly disagree	108 (11)
Moderately disagree	218 (22)
Moderately agree	482 (48)
Strongly agree	191 (19)
**Cost-effectiveness**	
Please indicate the degree to which you object (if at all), for moral reasons, to using cost-effectiveness data to determine which treatments will be offered to patients.	
No moral objection	457 (45)
Moderately moral objection	405 (40)
Strong moral objection	144 (14)

With respect to the MFQ30 measures, Cronbach alpha scores indicated fair-to-moderate internal consistency of the five moral foundations subscales (0.57 for the harm foundation, 0.62 for fairness and ingroup, 0.67 for authority, and 0.83 for purity). Overall, the harm foundation had the highest mean score (3.5), followed by fairness (3.3), authority (3.1), ingroup (2.8), and purity (2.7). Descriptive statistics for all items comprising the five subscales are included in Additional file 1.

**Figure 1:**
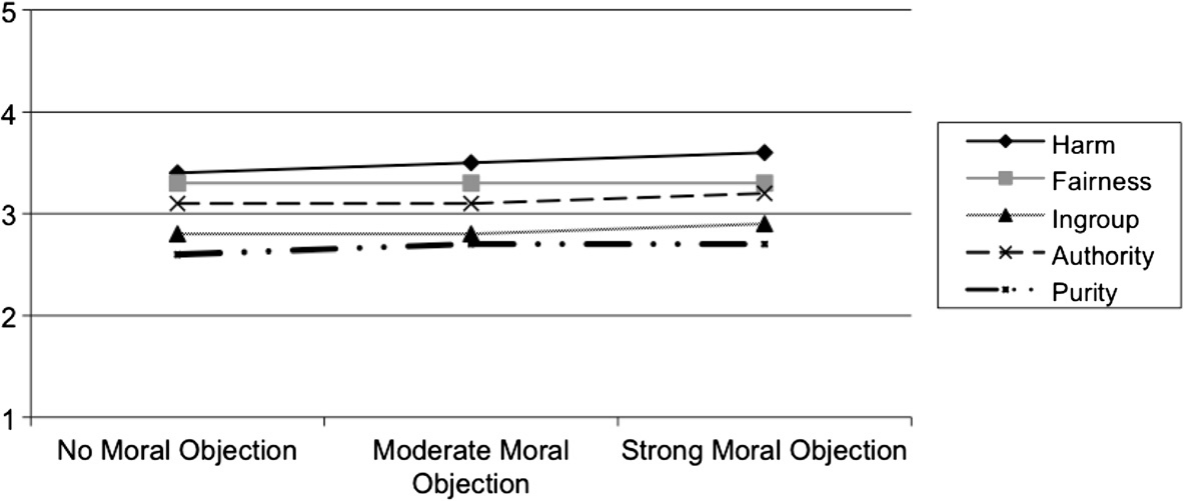
Moral foundations of physicians by the extent to which they object to using cost-effectiveness data.

**Figure 2:**
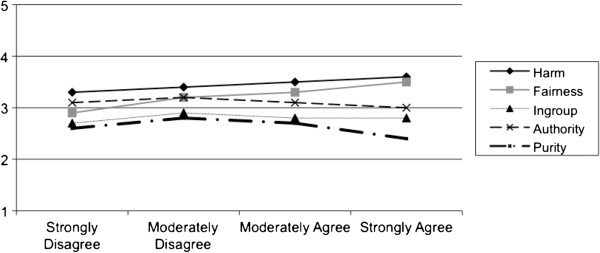
Moral foundations of physicians by whether they agree or disagree with cost-containment.

Figures 1 and 2 display the moral foundation scores of physicians by whether they object to using cost-effectiveness data and by the extent to which they agree or disagree with cost-containment, respectively. Physicians who strongly objected to the use of cost-effectiveness data had similar scores in all of the five foundations (Figure 1) (p-values for all pairwise comparisons > 0.05). Moral foundation scores did, however, differ with respect to physicians’ views regarding cost-containment. Physicians who strongly agreed with utilizing cost-containment measures had mean scores that were higher for the foundations of harm (3.6) and fairness (3.5) compared to the foundations of ingroup (2.8), authority (3.0), and purity (2.4) (p-values for all pairwise comparisons < 0.05).

Table 3 shows the associations between moral foundations scores and physicians’ judgments about cost. In unadjusted analyses, both harm and fairness were significantly associated with judgments about cost-containment. For every 1-unit increase in mean harm score (0–5), there was a 20% increased odds of agreeing with cost-containment (OR = 1.2 [1.0-1.4]). Similarly, every 1-unit increase in mean fairness scores was associated with 70% greater odds of agreeing with cost-containment. These associations were unchanged after adjusting for age, sex, region, and specialty. Harm ratings were also associated with moral objection to utilizing cost-effectiveness data in clinical decision-making. For every 1-unit increase in harm subscale scores (0–5), there was a 20% greater chance of objecting to cost-effectiveness analysis in clinical practice (OR = 1.2 [1.0-1.4]). However, that association did not remain significant after adjusting for demographics-related covariates. Fairness scores were not associated with judgments about using cost-effectiveness data in clinical practice. There was no association between ingroup, authority, or purity and cost-containment or cost-effectiveness judgments.

**Table 3:** Association between moral foundations subscales and judgments about cost-containment and using cost-effectiveness in clinical practice among 1032 US physicians

	**Cost-containment**		**Cost-effectiveness**	
	**I agree with limiting reimbursement for expensive drugs and procedures if that would help expand access to basic healthcare for those currently lacking such care.**		**I object to using cost-effectiveness data to determine which treatments will be offered to patients.**	
	**Unadjusted OR**	**Adjusted OR**	**Unadjusted OR**	**Adjusted OR**
	**(95% CI)**	**(95% CI)**	**(95% CI)**	**(95% CI)**
**Harm**	1.2 (1.0–1.4)*	1.2 (1.0–1.5)*	1.2 (1.0–1.4)*	1.2 (1.0–1.4)
**Fairness**	1.7 (1.4–2.0)*	1.7 (1.4–2.1)*	0.9 (0.8–1.1)	0.9 (0.7–1.0)
**Ingroup**	1.0 (0.8–1.1)	1.0 (0.8–1.2)	1.0 (0.9–1.2)	1.0 (0.9–1.2)
**Authority**	0.9 (0.8–1.0)	0.9 (0.8–1.1)	1.0 (0.9–1.2)	1.0 (0.9–1.2)
**Purity**	0.9 (0.8–1.0)	1.0 (0.9–1.1)	1.1 (1.0–1.2)	1.1 (1.0–1.2)

Table presents the odds ratios (and 95% confidence intervals) for agreeing with cost containment and objecting to using cost effectiveness data, by scores on each of the five moral foundations subscales. Odds ratios are for one-point increases in subscale score. Multivariable models are adjusted for age, sex, region, and specialty.

* p < 0.05.

## Discussion

In this national physician survey, we found that the harm and fairness intuitions of the MFQ30 – a measure of moral intuitions – were associated with physicians’ judgments about cost-containment, but not with their objection to using cost-effectiveness analysis in clinical decision-making.

These data drawn from a contemporary sampling of U.S. physicians offer some preliminary clues about why physicians may disagree on the role that cost and cost-information should play the contemporary health care. Much of the debate has centered around whether physicians should primarily act in each individual patient’s best interest, or make decisions that are in the best interest of society as a whole [12,16,24-26]. The tension between these two goals (best interest of individual patient versus best interest of society) was demonstrated in a recent study examining the beliefs of primary care physicians [18]. Beach et al. found that the majority (70%) of physicians agreed that the provider’s main responsibility is to each individual patient rather than to society, however a significant minority (30%) were either neutral or disagreed. Furthermore, this study reported that over half of the physicians who responded (53%) believed that it is the responsibility of society to provide everyone with the best available health care. Although the wording of these items did not directly use the word rationing, they bear on debates about physician roles in rationing. To the extent that physicians see their primary duty to individual patients, scenarios in which they are asked to circumscribe or limit that obligation for other obligations to society may prompt visceral reactions against the idea of rationing. Thus, these data may offer some hints behind the negativity surrounding the “R” word.

However, unlike previous studies we attempted to explain why physicians hold the judgments they do. Our attempt was only partially successful. Harm and fairness intuitions were independently associated with physicians’ judgments about cost-containment, yet, contrary to our hypothesis, these constructs were not independently associated with physicians’ objections to using cost-effectiveness analysis in clinical practice. The sources of variation in these judgments, beyond some simple demographic characteristics identified previously, [22] remain largely unexplained. It is possible that while a subset of physicians do not morally object to cost-effectiveness analysis conceptually, they may have concerns over particular cost-effectiveness data and how those data should be applied globally. Cost-effectiveness analysis can be limited by the quality of the data (for example specific biases in a particular trial), whether an appropriate control group was utilized, and whether the data is generated from direct measurement versus modeling of outcomes [27]. Furthermore, this type of enquiry requires an assessment of both cost and effectiveness. Measurements of these factors can vary considerably and affect the validity of the analysis.

These data also raise important questions about how best to accommodate prevailing professional norms of practicing U.S. clinicians in implementing cost-containment strategies. The majority of U.S. physicians object to using cost-effectiveness data to guide clinical decisions [22]. If the social intuitionist perspective is correct about how moral judgments are formed, advocates using cost to guide clinical decision-making should seek to persuade from within the mentality (intuition structure) of physicians with whom they disagree. Otherwise, mere education of physicians may not resolve the disagreement since the nature of the disagreement is as much a visceral as a cognitive one.

This study has important limitations. It is a cross-sectional assessment of opinions that may not be stable over time. In addition, responses may be biased due to non-response despite a 54% cooperation rate. The primary measures were cognitive pre-tested. Yet, in a sensitive area such as cost, how items are worded might influence physicians’ responses. For instance, using the word “rationing” might elicit a different reaction than the phrase “cost-containment”.

Notwithstanding these limitations, the results of our study suggest that efforts to enlist the support of physicians concerning cost-containment and cost-effectiveness in health care need to appeal to a range of moral intuitions that go beyond concerns regarding harm and fairness [22,23].

## Competing interests

The authors have no financial or non-financial competing interests to disclose in relation to this manuscript.

## Authors’ contributions

RMA conceived of the study, assisted with survey development, and drafted the manuscript. FAC assisted with survey development and helped to draft the manuscript. KMJ assisted with survey development, performed the statistical analyses, and helped to draft the manuscript. JCT conceived of the study, assisted with survey development, and helped to draft the manuscript. All authors read and approved the final manuscript.

## Supplementary Material

Additional file 1 **Characteristics of responses for each item used in calculation of the 5 moral foundations.** Response categories ranged from 0 (strongly disagree/not at all relevant) to 5 (strongly agree/extremely relevant). Click here for file
