# Supplementary material for: The moral psychology of rationing among physicians: the role of harm and fairness intuitions in physician objections to cost-effectiveness and cost-containment
Source: Philos Ethics Humanit Med. 2013 Sep 8;8:13. doi: 10.1186/1747-5341-8-13 (PMC3847359; doi:10.1186/1747-5341-8-13)
Supplement: Additional file 1 — Characteristics of responses for each item used in calculation of the 5 moral foundations. Response categories ranged from 0 (strongly disagree/not at all relevant) to 5 (strongly agree/extremely relevant). [file 1747-5341-8-13-S1.pdf]

APPENDIX A. Characteristics of responses for each item used in calculation of the 5 moral foundations. Response categories ranged from 0 (strongly disagree/not at all relevant) to 5 (strongly agree/extremely relevant)

Moral Foundation	Mean	SD	Range	No. missing	Cronbach's Alpha*
**Harm**					
Overall	3.5	0.8	0.8-5	19	0.57
Stem: Indicate your degree of agreement or disagreement with the following statements					
V32. Compassion for those who are suffering is the most crucial virtue.	4.1	1.0	0-5	19	
V38. One of the worst things a person could do is hurt a defenseless animal.	3.7	1.5	0-5	15	
V43. It can never be right to kill a human being.	2.5	1.9	0-5	20	
Stem: Indicate how relevant each of the following are in determining whether something is right or wrong. Whether or not someone…					
V48. Suffered emotionally.	3.3	1.2	0-5	20	
V54. Cared for someone weak or vulnerable.	3.3	1.3	0-5	26	
V59. Is cruel.	4.2	1.1	0-5	23	
**Fairness**					
Overall	3.3	0.7	0. 7-5	19	0.62
Stem: Indicate your degree of agreement or disagreement with the following statements					
V33. When the government makes laws, the number one principle should be ensuring that everyone is treated fairly.	3.7	1.4	0-5	22	
V39. Justice is the most important requirement for a society.	3.7	1.0	0-5	17	
V44. I think it's morally wrong that rich children inherit a lot of money while poor children inherit nothing.	1.2	1.4	0-5	18	
Stem: Indicate how relevant each of the following are in determining whether something is right or wrong. Whether or not someone…					
V49. Was treated differently than others.	3.3	1.2	0-5	20	
V55. Acts unfairly.	3.6	1.2	0-5	23	

V60. Denies others their rights.	4.3	1.0	0-5	26	
**Ingroup**					
Overall	2.8	0.8	0.5-5	19	0.62
Stem: Indicate your degree of agreement or disagreement with the following statements					
V34. I am proud of my country's history.	3.9	1.4	0-5	17	
V40. People should be loyal to their family members, even if they have done something wrong.	2.8	1.5	0-5	19	
V45. It is more important to be a team player than to express one's self.	2.0	1.4	0-5	18	
Stem: Indicate how relevant each of the following are in determining whether something is right or wrong. Whether or not someone…					
V50. Shows love for his or her country	2.2	1.5	0-5	22	
V56. Did something to betray his or her group	3.2	1.3	0-5	28	
V61. Shows a lack of loyalty.	2.9	1.3	0-5	26	
**Authority**					
Overall	3.1	0.8	0.7-5	20	0.67
Stem: Indicate your degree of agreement or disagreement with the following statements					
V35. Respect for authority is something all children need to learn.	4.3	0.9	0-5	17	
V41. Men and women each have different roles to play in society.	3.0	1.6	0-5	21	
V46. If I were a soldier and disagreed with my commanding officer's orders, I would obey anyway because that is my duty.	2.9	1.5	0-5	14	
Stem: Indicate how relevant each of the following are in determining whether something is right or wrong. Whether or not someone…					
V51. Shows a lack of respect for authority.	2.9	1.4	0-5	23	
V57. Conforms to the traditions of society.	2.2	1.3	0-5	26	
V62. Causes chaos or disorder.	3.4	1.3	0-5	23	
**Purity**					
Overall	2.7	1.2	0-5	22	0.83
Stem: Indicate your degree of agreement or disagreement with the following statements					
V36. People should not do things that are disgusting, even if no one is harmed.	3.0	1.7	0-5	23	

V42. I would call some acts wrong on the grounds that they are unnatural.	2.4	1.7	0-5	29	
V47. Chastity is an important and valuable virtue.	2.9	1.6	0-5	33	
Stem: Indicate how relevant each of the following are in determining whether something is right or wrong. Whether or not someone…					
V52. Violates standards of purity and decency.	2.9	1.5	0-5	28	
V58. Does something disgusting.	2.3	1.5	0-5	26	
V63. Acts in a way that God would approve of.	2.6	1.9	0-5	35	

*Raw Cronbach's alpha scores were utilized.
